# Visual acuity of budgerigars for moving targets

**DOI:** 10.1242/bio.058796

**Published:** 2021-09-03

**Authors:** Sandra Chaib, Juliane Gaviraghi Mussoi, Olle Lind, Almut Kelber

**Affiliations:** Lund Vision Group, Department of Biology, Lund University, 223 62 Lund, Sweden

**Keywords:** Bird vision, Visual resolution, Dynamic acuity, Target detection, Object detection

## Abstract

For a bird, it is often vital to visually detect food items, predators, or individuals from the same flock, i.e. moving stimuli of various shapes. Yet, behavioural tests of visual spatial acuity traditionally use stationary gratings as stimuli. We have behaviourally tested the ability of budgerigars (*Melopsittacus undulatus*) to detect a black circular target, moving semi-randomly at 1.69 degrees s^−1^ against a brighter background. We found a detection threshold of 0.107±0.007 degrees of the visual field for a target size corresponding to a resolution of a grating with a spatial frequency of 4.68 cycles degree^−1^. This detection threshold is lower than the resolution limit for gratings but similar to the threshold for stationary single objects of the same shape. We conclude that the target acuity of budgerigars for moving single targets, just as for stationary single targets, is lower than their acuity for gratings.

## Abstract (precis)

**Summary:** Movement does not improve detection: The detection threshold of budgerigars (*Melopsittacus undulatus*) for moving dark circular targets is similar to the detection threshold for stationary but otherwise similar targets.

## INTRODUCTION

Vision is undoubtedly one of the primary senses of birds (Martin, 2017). The excellent colour vision (Kelber, 2019), as well as high spatial (Fischer, 1969; Reymond, 1985) and temporal resolution (Boström et al., 2016; Potier et al., 2020) of some species, are among the best in the animal kingdom.

In psychophysics, the common way to measure visual spatial acuity is determining the sinusoidal or square-wave grating of the highest spatial frequency that the eye can resolve. Assuming that the retinal mosaic limits spatial resolution of vision, the acuity limit is reached when adjacent black and white bars in a square-wave grating fall on the receptive fields of neighbouring sampling units (e.g. photoreceptors, or ganglion cells) in the retina (Land and Nilsson, 2012). This is a useful method to get a standardized comparative measurement of the resolving power of the eye (Barten, 1999; De Valois and De Valois, 1991). However, gratings rarely exist in nature, and thus, to understand how spatial resolution influences visually guided behaviour in an ecological context, other measures may be more interesting. For example, when asking at what distance a passerine can detect a conspecific, or a raptor can spot its prey, target acuity – which we define as the detection threshold, or the minimal resolvable angle, for small or distant single objects, might give a more relevant answer (Chaib et al., 2019). In order to compare grating and target resolution, we assume that the size of the target (in degrees [deg] of visual field) equals half a cycle (one black or one white stripe) of a square wave grating.

Many animals, including humans, have been shown to possess higher acuity for single targets than for gratings (Ehrenhardt, 1937; Hecht et al., 1947; Vallet and Coles, 1993). Humans can resolve gratings with a spatial frequency of around 60 cycles deg^−1^, meaning that a single black or white stripe in the grating is 0.0083 deg wide. However, we can detect a single black line on a uniformly bright background, for instance a rope in front of the sky, even when it is only 0.00012 deg wide (Hecht et al., 1947), thus about 70 times narrower. Thus, target acuity is theoretically limed by contrast sensitivity, while grating acuity is limited by the resolving power of the retina (O'Carroll and Wiederman, 2014). In a recent study, we showed that this was not the case for budgerigars (*Melopsittacus undulatus*, Shaw 1805) that have similar acuity for single targets and gratings (Chaib et al., 2019). Budgerigars can resolve gratings with 7.7 to 10 cycles deg^−1^, in which one black or white stripe subtends 0.05 to 0.065 deg of their visual field (Haller et al., 2014; Lind and Kelber, 2011; Lind et al., 2012), while they can just detect single targets of between 0.065 and 0.098 deg size, depending on the luminance profile of the target (Chaib et al., 2019). The main reason for this difference between humans and birds is presumably the birds' lower sensitivity for achromatic contrast (Ghim and Hodos, 2006; Haller et al., 2014; Harmening et al., 2009; Hirsch, 1982; Hodos et al., 2002; Lind et al., 2013, 2012; Orlowski et al., 2012; Potier et al., 2018; Reymond and Wolfe, 1981). Birds require around 10% Michelson contrast to discern gratings, while humans need less than 1% (De Valois, et al., 1974). A high contrast target smaller than the resolution limit determined for gratings, will be perceived as having lower and lower contrast to the background, with decreasing size. For a bird the detection threshold will be reached for a larger target compared to for a human.

Many natural targets that are vital for a bird, such as a soaring falcon or a flying prey animal, are not stationary but rather dynamic. Moving visual objects are not necessarily perceived in the same way as stationary objects. The movement of an object relative to the background can break camouflage (Hall et al., 2013) or catch the viewer's attention (Richard and Shawn, 2003; Rushton et al., 2007), thereby making the object more salient and potentially lower the detection threshold. In humans, visual acuity is mostly impaired as a function of movement (Brown, 1972; Lewis et al., 2011), but under some circumstances it can also be improved. For example, peripheral visual acuity is slightly improved by slow target motion (Brown, 1972).

To our knowledge, the effect of motion on acuity and contrast sensitivity of birds has only been investigated with gratings. The contrast sensitivity of budgerigars is higher for horizontally drifting than for stationary achromatic gratings (Haller et al., 2014). For high spatial frequency (6.5 cycles deg^−1^) gratings, a velocity of 1.4 deg s^−1^ almost doubles contrast sensitivity for budgerigars. While Tyrrell et al. (2014) found that starlings (*Sturnus vulgaris*) were not very likely to visually fixate a stationary or a moving black dot, the effect of movement on the detection threshold of single targets has not previously been investigated.

During our previous experiment (Chaib et al., 2019) it was surprisingly difficult to train budgerigars to the task of detecting stationary single targets. If the unexpectedly low visual acuity for stationary targets was influenced by the lack of motivation from the birds, this could potentially be overcome by movement of the target (Pratt et al., 2010; Richard and Shawn, 2003). As a result of this assumption, our expectation was that budgerigars could detect smaller moving targets than stationary targets.

## RESULTS AND DISCUSSION

**Fig. 1.**
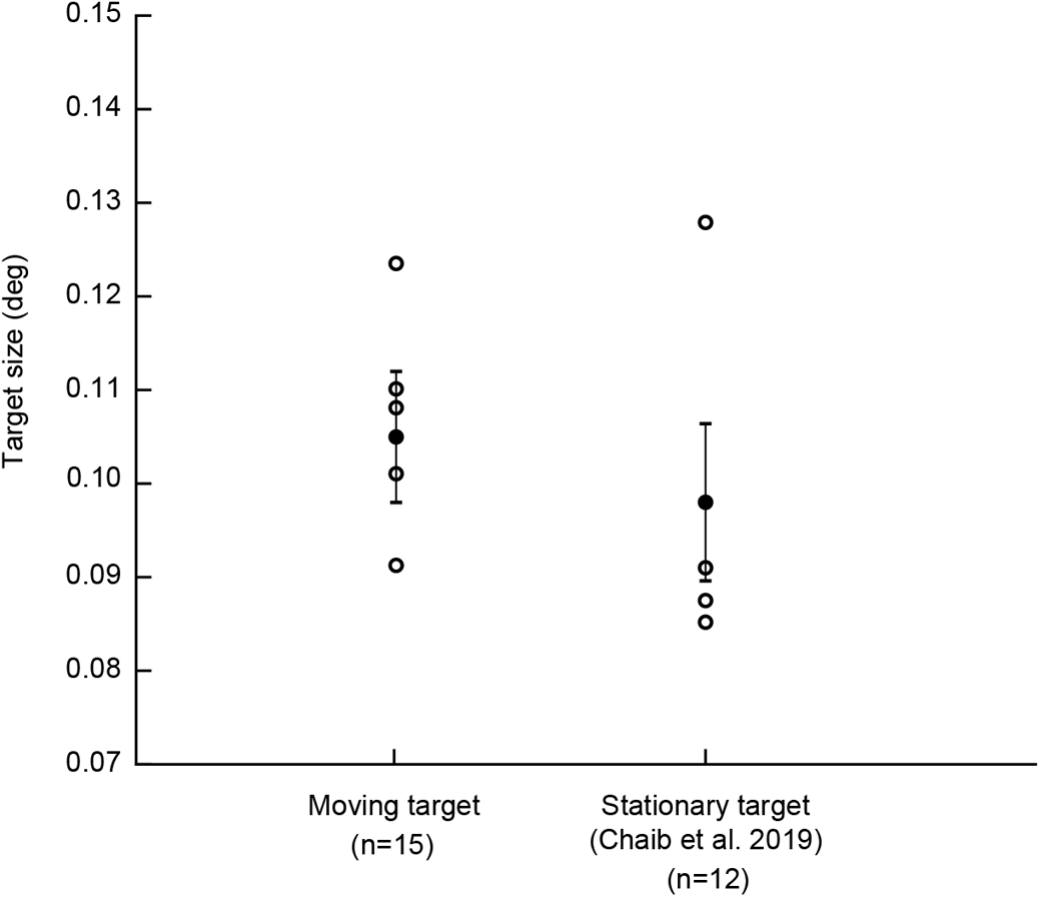
**Detection thresholds for moving, as well as stationary, single targets.** Empty circles represent the thresholds for individual birds and filled circles represent means for all birds in the experiment. Error bars represent s.d.

Five of seven budgerigars learned to associate the presence of the target with a reward. They were able to detect a moving black target with a diameter subtending 0.107±0.007 deg (mean±sd) of their visual field (Fig. 1), corresponding to a black-and-white grating with a spatial frequency of 4.68±0.32 cycles deg^−1^ (in which a black and a white stripe subtend 0.214 deg). The bird with the highest acuity could detect a target subtending 0.091 deg of the visual field (5.48 cycles deg^−1^), while the bird with the lowest acuity could detect a target subtending 0.124 deg (4.04 cycles deg^−1^). Just as for stationary targets, the detection threshold for single black targets was lower than expected on the basis of grating acuity (7.7 to 10 cycles deg^−1^; Chaib et al., 2019; Haller et al., 2014; Lind and Kelber, 2011).

We knew from our earlier study that budgerigars have a detection threshold of 0.098±0.008 deg, corresponding to 5.1±0.45 cycles deg^−1^, for a stationary target of the same shape and contrast to the background as the moving target (Chaib et al., 2019). Three birds participated in both experiments. To find out whether these two detection thresholds differ significantly, we fitted a linear mixed-effects model with random intercepts to the combined data with experiment type (moving target versus stationary target) as a fixed effect and individual birds as a random effect. We compared this model to a reduced model excluding the experiment type (fixed effect) and did not find a significant effect of experiment type on the detection threshold (χ^2^=0.74, d.f.=1, *P*=0.39, AIC full model: −114.9, AIC reduced model: −116.2). This indicates that, contrary to our expectation, the detection threshold for moving targets is not significantly different from the detection threshold for stationary targets.

We have previously calculated that a budgerigar, with a target acuity of around 0.1 deg of the visual field, would be able to spot a soaring brown falcon *Falco berigora* from a distance of 85 m (Chaib et al., 2019). We conclude from the present study that for a falcon moving at 1.69 deg s^−1^ the distance would be roughly the same. However, a speed of 1.69 deg s^−1^ would correspond to a groundspeed of 2.5 m s^−1^ from this distance, which is considerably lower than the soaring flight speed measured in other falcons (Cochran and Applegate, 1986; Rosén and Hedenström, 2002). In this study, we chose a retinal speed of the target which had previously shown to increase contrast sensitivity in budgerigars (Haller et al., 2014). This does not rule out that an ecologically more relevant speed, similar to that of a flying raptor, might yield a different result.

We hoped that a moving target would make it easier and more intuitive for the birds to detect the target and thus to learn the task, but this was not the case. This may be related to the findings by Tyrrell et al. (2014) that starlings were not more likely to visually fixate a randomly moving black dot compared to a stationary black dot. Interestingly the starlings trained by Tyrrell et al. (2014) only fixated the dot in 25% of the trials. Visually relevant stimuli, like a moving mealworm or a Harris's hawk (moving or stationary) were more likely to be fixated by the birds than the dot (Tyrrell et al., 2014). The fact that a starling is more likely to fixate on a stationary image of a hawk compared to a moving dot suggests that stimulus shape might be a greater indicator of importance than movement. However, besides differing in shape, these stimuli also differed in size, contrast, and movement type making it difficult to separate shape as an exclusive factor (Tyrrell et al., 2014). Moreover, in studying visual acuity, using elaborate targets like raptor silhouettes provides difficulties in quantifying the size of the target and thus comparing to other measures of spatial vision.

Another relevant factor may be the stimulus position. Chickens react to a black round target moving in a straight line above their head by predator avoidance response, including visual fixation (Hébert et al., 2019). The position of the stimulus, as well as the pattern of movement, likely have an impact on the relevance of the stimulus for the bird. Birds which are naturally exposed to aerial predators, like budgerigars, starlings and chickens, might be prone to fixate a dorsal straight moving target. A randomly moving target in the horizontal field of view, on the other hand, might be of less importance to ground foraging birds, although starlings occasionally catch insects in the air (Tinbergen, 1981). It is possible that birds of prey, or birds specialized in hawking, are more prone to pay attention to small unidentifiable moving targets. However, Harris's hawks also have proved difficult to condition to small moving targets (Simon Potier, Lund University, personal communication).

Experiments in optimal foraging suggest that birds will spend more time foraging by walking (a low-cost way of travel) with a low yield compared to foraging by flying (a high-cost way of travel) with a high yield (Bautista et al., 2001). With this in mind, we had trained the birds to walk instead of fly in a smaller experimental arena. Our expectation was that the birds would be able to do more trials per session for a smaller food reward. However, we did not experience a great difference in the birds' willingness to participate in the experiment compared to in previous experiment when the birds were flying.

To conclude, the target acuity of budgerigars is not better for moving targets than for stationary targets. Budgerigars do not instinctively visually fixate randomly moving black targets in the frontal or lateral visual field. It is possible that the position of the target might be of relevance and that a budgerigar might react differently to a dorsally presented target. An interesting future direction would be to investigate the moving target acuity in birds foraging on flying prey, like insects or small birds.

## MATERIALS AND METHODS

### Animals

Three female and four male budgerigars participated in the experiment. Three of these birds had also participated in a previous experiment with stationary targets (Chaib et al., 2019). The birds were fed a millet-based seed mix adapted for parakeets, vegetables and fruit except for experimental days when they received the seed mix only as a reward in the experiment, complemented by vegetables and fruit in the home cage. The birds participated in the experiments four consecutive days a week and rested for three days. All experiments were performed following Swedish legislation, under the permit M111-14 from the local authority for animal ethics.

### Experimental setup

**Fig. 2.**
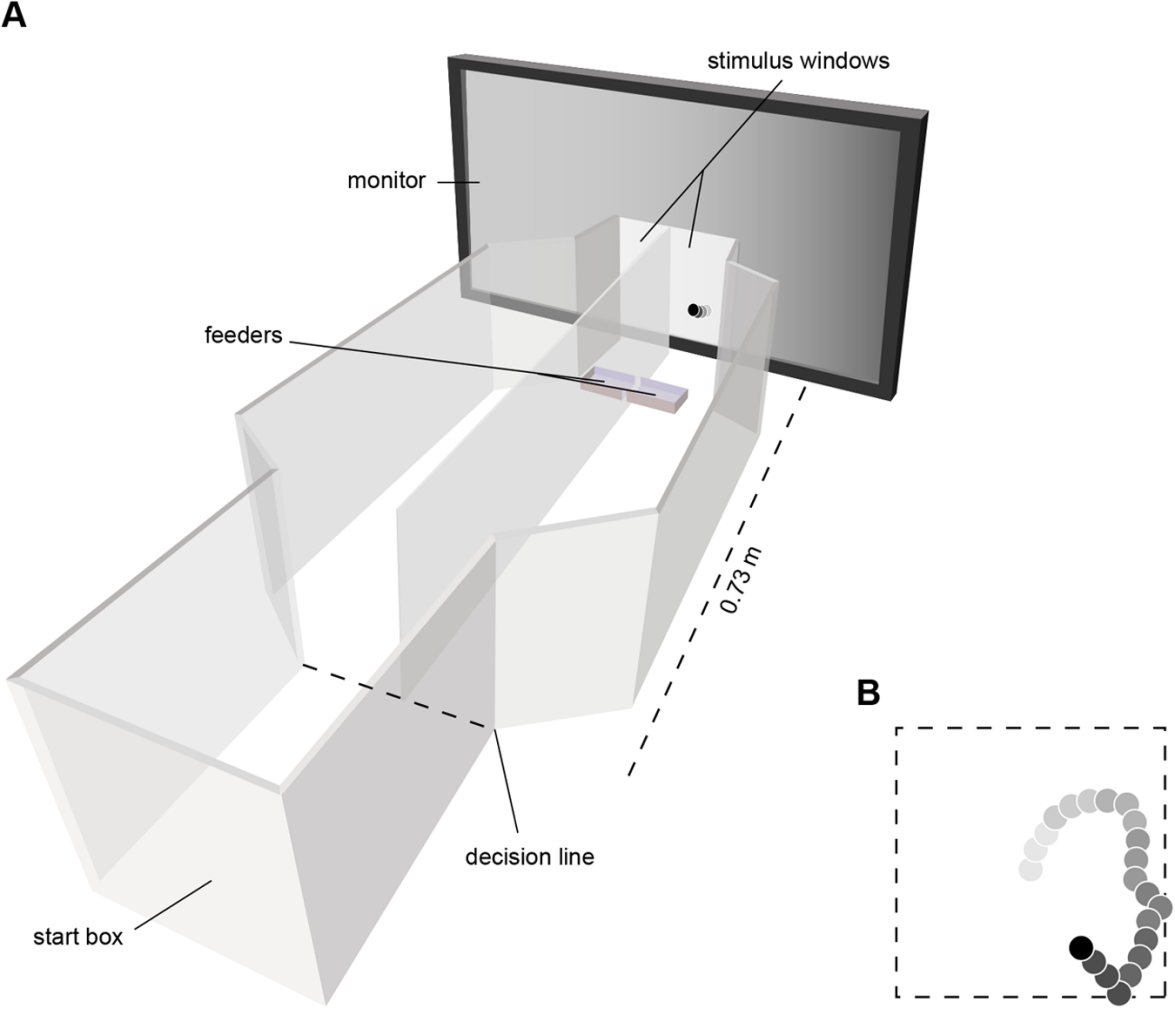
**The experimental setup and stimulus.** (A) The experimental setup. At the start of each trial, the bird was positioned in the start box viewing the monitor. When the target was displayed in one of the stimuli windows, the bird would make its choice by entering one of the corridors. The part of the monitor not visible in the stimuli windows were dark throughout the trials. The experimental arena was covered by a lid of transparent polyacrylic and a black fabric surrounded the sides of the setup (not seen in the figure). (B) An example of a target trajectory. The dashed line represents the invisible boundary in which the target centre moved.

The experiments were performed in a y-maze with a removable top constructed of opaque polyacrylic sheets. A 15 cm wide, 20 cm long and 20 cm high compartment, the *start box*, would hold the bird at the start of each trial (Fig. 2A). The start box was open to two 73 cm long, 15 cm wide and 20 cm high corridors leading to two stimulus windows, each 15 cm high and 7 cm wide corresponding to 11.6×5.5 deg of visual angle as seen from the *decision line* (the boundary between the start box and the corridors; Fig. 1A). A monitor (32WL30MS, LG, Seoul, South Korea) positioned behind the stimulus windows displayed the stimuli (Fig. 1A). A feeder was positioned at the end of each corridor. Each feeder was connected to a food dispenser (Lind, 2016) by a plastic tube (not shown in the figure).

### Stimuli

The rewarding stimulus consisted of a black dot (0.23 cd/m^2^), the *target*, moving in a semi-random manner on a bright grey background (140 cd/m^2^; >99% contrast). The direction in which the target moved for every new frame was normally distributed around the previous direction of travel. This way, the target had an erratic movement, although with smooth turns. The trajectory of the target centre never moved outside an area subtending 0.72×0.72 cm in the stimulus window and 0.56×0.56 deg of visual angle, as seen from the decision line. When the target reached the invisible boundary, the direction was reversed (Fig. 2B). It has been shown that budgerigars have higher contrast sensitivity for drifting than for stationary gratings (Haller et al., 2014). Thus, to obtain the most favourable conditions in the experiment, we set the target speed to 1.69 deg s^−1^ as seen from the decision point of the bird. This is close to the speed at which maximal contrast sensitivity was measured in the study by Haller et al. (2014). The unrewarding stimulus consisted of the same bright grey background as the rewarding stimulus but lacked the target. The stimuli were created in Matlab using the Psychophysics Toolbox extensions (Brainard, 1997; Pelli, 1997; Kleiner et al., 2007).

### Experimental procedure

After an auditory start signal (three short consecutive tones), the rewarding stimulus, with the moving target, appeared in one of the stimulus windows – either the left or the right. The bird was trained to enter the corridor leading to the stimulus window presenting the target. When the bird made a correct choice, entering the corridor leading to the rewarding stimulus, a high pitch signal would sound, and a few seeds would be delivered into the feeder in that corridor. When the bird made an incorrect choice, and entered the corridor where no target was present, a low pitch signal would sound, and the stimuli would disappear from the stimuli windows. In both cases, a new trial started after the bird had returned to the start box and faced the corridors.

**Fig. 3.**
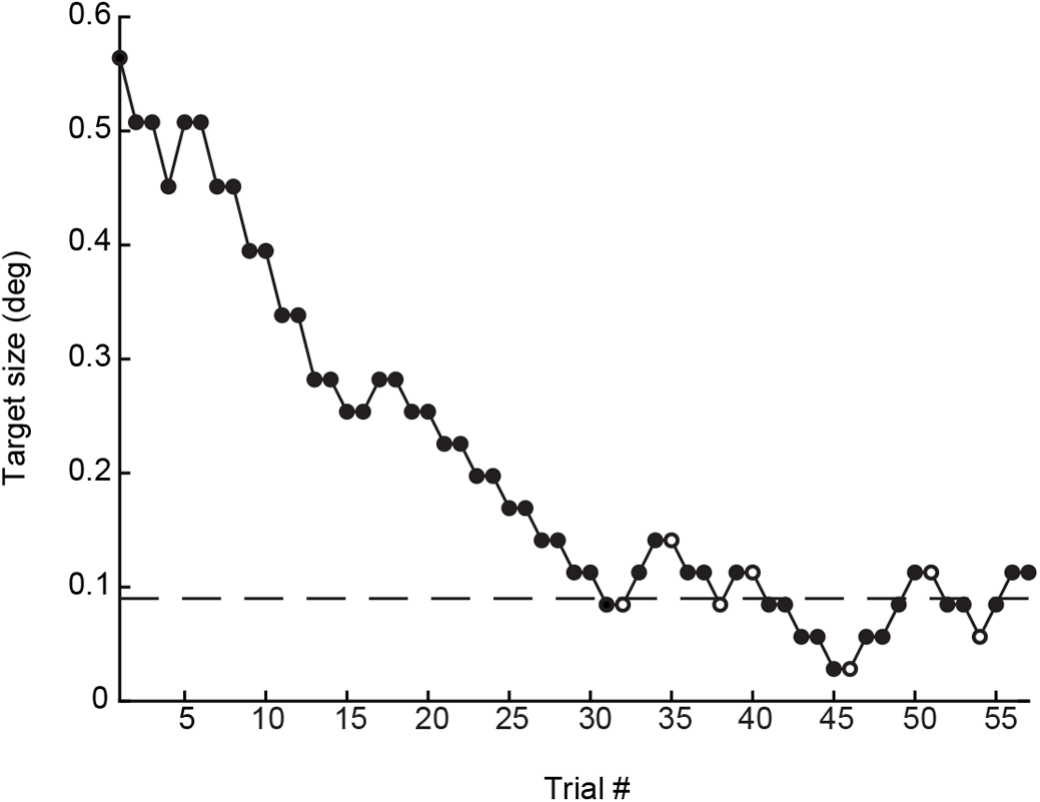
**An example of an adaptive staircase.** Empty circles represent reversal points and the dashed line is the detection threshold of the session, calculated as the mean of the reversals in the last 25–30 trials. The example is taken from ‘female 2’ (Fig. S1D). All test sequences included in the analysis of the experiment can be found in the Supplementary Material (Fig. S1A–E).

In the initial training sessions only the largest targets, with diameters of 1.44 and 0.71 cm or 1.13 and 0.56 deg of the visual field, were used. Once a bird had learned to choose the correct side in the y-maze, we started the staircase sessions, in which the size of the target was changed following an adaptive 1-up/2-down staircase procedure (Levitt, 1971; Fig. 3). In the staircase sessions, the initial target size was 0.56 deg of the visual field, which is well above the detection threshold of the birds (Chaib et al., 2019). Target size would decrease after two consecutive correct choices, but increase again after one incorrect choice, until target size fluctuated around the level at which the probability of a decrease of target size equals the probability of an increase of target size. This level corresponds to the point on a psychometric function where the probability of making a correct choice is 70.7% (Levitt, 1971). The staircase step sizes were ±0.056 deg (of the diameter of the target) above a target size of 0.282 deg and ±0.028 deg below this size. Each test session consisted of 45–60 trials depending on the motivation of the bird. Consistent with our experience from previous experiments using stationary targets, the birds improved their detection threshold during the first three to five training sessions until they reached a plateau (Chaib et al., 2019). If a bird did not improve over three sessions in a row, we concluded that this represented its maximal performance and ended the experiment.

### Analysis

We analysed the data from the last three sessions for each bird, which are the sessions after the bird reached the performance plateau. The thresholds were estimated by averaging the reversal points, the values in the staircase where the curve slope changes direction (Fig. 3), of the last 25–30 trials (depending on the length of the session) in each of the three sessions. We used an even number of reversal points for each test session to avoid any estimation bias (Levitt, 1971). The individual thresholds for each test session obtained this way were compared to the detection thresholds for stationary targets that had been determined in a previous experiment. A linear mixed-effects model with random intercept was fitted to the pooled data from both experiments, including birds participating in both experiments as well as birds only participating in one of the experiment. The model included with experiment type (stationary target or moving target) as a fixed effect and bird identity as a random effect, using the lmerTest package (Kuznetsova et al., 2017) in RStudio (v. 1.1.463; R Studio Team, 2016). This model was compared to a reduced model, excluding the fixed effect of experiment type, with a log-likelihood ratio test. Additionally, the two models were compared by their Akaike information criterion (AIC) values.

## Supplementary Material

Supplementary information
