# Supplementary material for: Visual acuity of budgerigars for moving targets
Source: Biol Open. 2021 Sep 3;10(9):bio058796. doi: 10.1242/bio.058796 (PMC8473842; doi:10.1242/bio.058796)
Supplement: Supplementary information [file biolopen-10-058796-s1.pdf]

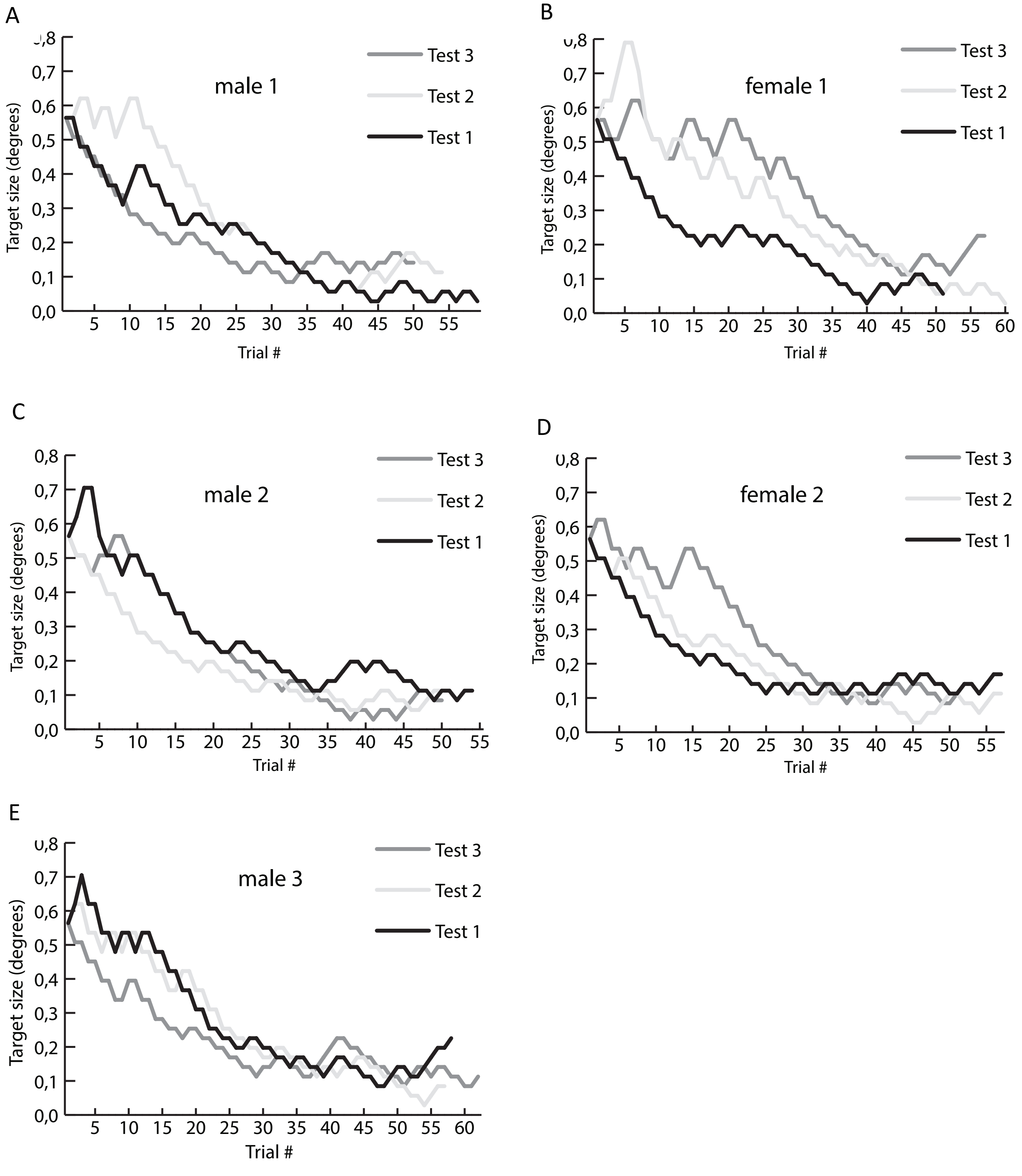

Fig. S1. Results from the moving target acuity test following an adaptive 2-down/1-up procedure. Fig A-E show the three test sessions from each of the five birds included in the analysis of the experiment.
